# Traction MRI of the Elbow: Age-Based Effects and Implications

**DOI:** 10.3390/diagnostics14192165

**Published:** 2024-09-28

**Authors:** Sho Kohyama, Kazuhiro Ikeda, Yoshikazu Okamoto, Yuichi Yoshii

**Affiliations:** 1 Department of Orthopaedic Surgery, Kikkoman General Hospital, Noda 278-005, Chiba, Japan; sho_kohyama_1025@yahoo.co.jp; 2 Department of Orthopaedic Surgery, University of Tsukuba, Tsukuba 305-8575, Ibaraki, Japan; 3 Department of Diagnostic Imaging, Tohoku University School of Medicine, Sendai 980-8575, Miyagi, Japan; 4 Department of Orthopaedic Surgery, Tokyo Medical University Ibaraki Medical Center, Ami 300-0395, Ibaraki, Japan

**Keywords:** magnetic resonance imaging, traction, elbow, articular cartilage, age

## Abstract

Background/Objectives: We previously reported that traction magnetic resonance imaging (MRI) of the elbow without arthrography increases the width of the radiocapitellar joint (RC) and improves articular cartilage visibility. However, the effects of axial traction on different age groups have not yet been evaluated. We hypothesized that the effect of traction would decrease as the participants’ age increased. Methods: We enrolled 30 healthy volunteers, ten each in their 20s, 30s, and 40s. The male-to-female ratio in each age group was 1:1. Elbow MRI was performed without traction and with 3, 5, and 7 kg axial traction. We evaluated joint space width (JW), humeral articular cartilage visibility, and intraprocedural pain/discomfort. We measured JW and cartilage visibility at the RC and the lateral and medial thirds of the ulnohumeral joint. Results: The older age groups exhibited narrower JWs without traction. Axial traction increased the JW and improved the visibility of the RC in all age groups. No significant differences were observed in the ulnohumeral joint’s lateral or medial thirds, but pain and discomfort increased with heavier traction weights. Conclusions: For participants in their 20s and 30s, axial traction of 3 kg seemed appropriate, while 7 kg traction was considered for those in their 40s.

## 1. Introduction

Effective treatment of articular cartilage injuries requires an accurate assessment of the cartilage to determine suitable intervention strategies. Several conditions can lead to such injuries, including elbow osteoarthritis (OA), capitellar osteochondritis dissecans, trochlear and trochlear notch chondromalacia [1,2,3,4,5,6], osteochondral fractures, and cartilage damage to the capitellum associated with posterolateral rotational instability [7], radial head fractures [8], and collagen diseases such as rheumatoid arthritis [9]. The intricate and compact anatomy of the elbow often complicates the precise evaluation of articular cartilage using magnetic resonance imaging (MRI) [10]. This complexity is heightened by the proximity of the articular cartilage surface of the capitellum to the opposing radial head, where most cartilage injuries occur [10]. Due to the similar MRI signal intensities of these opposing articular cartilages [11], accurately delineating the cartilage outlines for a thorough lesion assessment can be challenging [12]. Precise intra-articular imaging is essential for diagnosing the condition and pathology of articular cartilage as well as assessing the extent of synovitis and bone edema. Enhanced MRI enables more precise lesion evaluation, leading to more personalized treatment.

In earlier studies on this topic, we reported that using 7 kg of axial traction during elbow MRI significantly widened the radiocapitellar joint (RC) space width and improved the visibility of the articular cartilage without requiring a contrast medium [13]. We also evaluated the effects of different traction weights (3, 5, and 7 kg) and determined that 3 kg were optimal for elbow MRI, as this balanced the benefits of the procedure [14]. Additionally, we found no differences in the effectiveness of axial traction between female and male participants [14]. However, it is not reasonable to state that axial traction has the same effect on all age groups, from young adults to older adults. In our previous studies, the subject groups comprised participants from different age groups. Therefore, to date, the impact of axial traction on different age groups has not been evaluated.

In this study, we hypothesized that the effect of traction would decrease as the participants’ age increased. Therefore, we evaluated the effects of axial traction in different age groups. This study aimed to clarify the effects of axial traction using three different traction weights in three different age groups.

## 2. Materials and Methods

### 2.1. Recruitment of Volunteers

This study was approved by the Institutional Review Board of Kikkoman General Hospital (approval number: KC-H24) and conducted in accordance with the Declaration of Helsinki (2013 revision). We enrolled 30 healthy volunteers from hospital staff, with ten participants each in their 20s, 30s, and 40s, all of whom had no prior elbow injuries or current elbow-related symptoms. The male-to-female ratio was 1:1. Following institutional review board approval, the first author recruited volunteers after announcing the study. All participants provided written informed consent and underwent clinical examinations conducted by the first author, who has over 15 years of experience as an elbow surgeon. The examinations assessed the volunteers’ range of motion, instability, and tenderness on both the lateral and medial sides of the elbow. Volunteers were excluded if any abnormalities were found; however, none exhibited such findings, allowing them to be included in the study.

### 2.2. Image Acquisition

Between April 2021 and November 2023, we performed MRI scans using a 1.5-Tesla system (Achieva, Philips Medical Systems©, Best, The Netherlands) and an eight-channel coil (Philips Medical Systems©, Best, The Netherlands). We applied a three-dimensional T1-fast echo with water excitation for cartilage (WATS-c), a field of view of 130 × 130 × 60 mm, a matrix of 256 × 195, slice thickness and gap of 0.4 mm, a repetition time of 20 ms, a flip angle of 25°, and an echo time of 8.4 ms [13,14]. The right elbow, which was the dominant side of all volunteers, was the focus of our investigation. Each image acquisition lasted for 3 min 52 s. Additional time was required to adjust the traction weight between the scans, resulting in a total imaging duration of approximately 20 min per volunteer. If a volunteer moved involuntarily during the scan, the sequence was repeated to avoid motion artifacts.

### 2.3. Traction MRI

**Figure 1:**
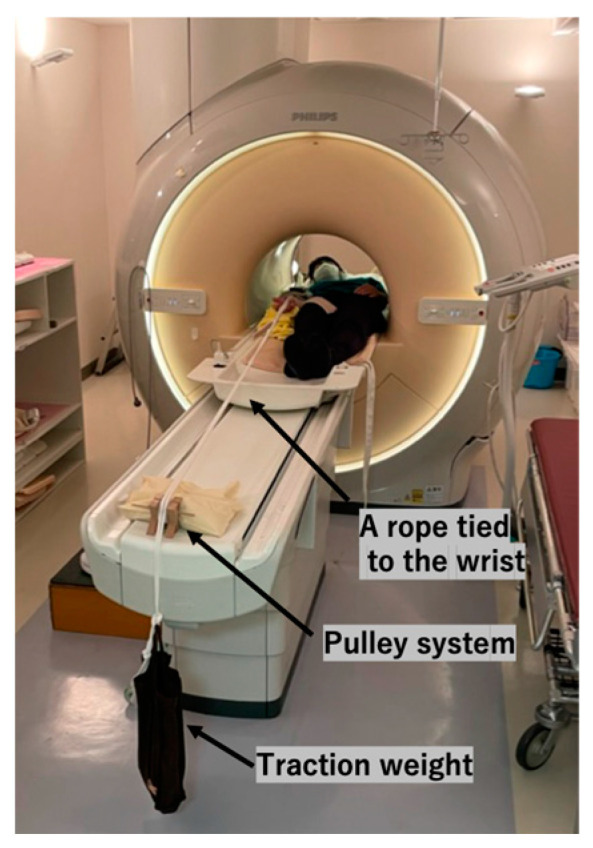
Traction setup. We secured a rope to the volunteer’s wrist, with a weight fastened to the opposite end. The rope was then suspended over the table’s edge using a pulley system.

First, we performed an MRI without traction. Subsequently, a traction MRI was conducted using a setup identical to that described in the literature [13,14]. We confirmed that the elbow was in full extension during imaging and secured the position with two bands. We ensured not to hyperextend the elbow during the setup. Simultaneously, we instructed the participants to keep their forearm in a supinated position as far as possible without discomfort, which meant not necessarily in full supination. A sponge and rope were wrapped around the right wrist of each participant. We attached the weight to the opposite end of the rope, which was suspended over the side of the MRI table using a pulley system (Figure 1). We began with axial traction using 3 kg, then increased the weight to 5 kg, and finally, to 7 kg. We did not rearrange the positioning of the elbow when the traction weights were applied.

### 2.4. Image Analysis

Using MR, we assessed the joint space width (JW), humeral articular cartilage visibility (HACV), and intra- and inter-rater reliability for evaluating JW and HACV. Since most elbow articular cartilage injuries occur on the humeral articular surfaces [5,6,7,8], our investigation focused solely on the humeral articular cartilage. The JSW was defined, according to our previous studies [13,14], as the distance from the surface of the humeral articular cartilage to the opposing articular surfaces of the examined joint.

Two elbow surgeons, each with more than 10 years of clinical experience, independently reviewed all MR images under the guidance of a musculoskeletal radiologist (Y.O.) with more than 20 years of clinical experience. We utilized Osirix MD (version 14.0 Pixmeo©, Bernex, Switzerland) for image interpretation and to obtain multiplanar reconstructed images. The first author reconstructed all the sagittal and coronal images parallel to the longitudinal axis of the humerus. During their assessments, the examiners could freely magnify and adjust the grayscale contrast of the images to optimize the structural visualization. We randomly numbered All MR scans to minimize the potential examiner bias. Recognizing the possibility of examiner-related measurement errors even with MRI, each examiner measured each parameter twice to evaluate intraobserver correlations. The second set of assessments was performed at least two weeks after the first.

### 2.5. The JW Measurements

We evaluated the JSW at the RC, the lateral third of the ulnohumeral joint (LUH), and the medial third of the ulnohumeral joint (MUH). The JW measurement points and HACV grading procedures were based on our previous study [13,14], with examiners strictly adhering to these definitions for consistency. Specifically, on the coronal image, where the radial head appeared the largest, we identified the midpoint of the radial head and the lateral and medial thirds of the ulna. The JWs were measured on sagittal images corresponding to these points. For the RC, the JW was measured on a vertical line extending proximally from the center of the radial head, which was determined on a sagittal image passing through the midpoint of the radial head on a coronal image. We identified the horizontal bisector of the humerus relative to its longitudinal axis on sagittal images, passing through the lateral and medial thirds of the ulna. We then measured the JW at the LUH and MUH along the lines extending distally from the bisector.

We evaluated the differences in the JW values with and without traction across different age groups: 20s, 30s, and 40s. In addition, we assessed the effects of traction within each age group.

### 2.6. The HACV Assessments

On the sagittal images where we measured the JWs, we assessed the HACV using the same three-point scale as in our previous study [13,14]. Visibility was classified as poor when less than 50% of the humeral articular cartilage outline was visible in the area facing the opposite articular cartilage. Visibility was rated as intermediate when 50% to 99% of the humeral articular cartilage outline was visible. It was considered complete when 100% of the humeral articular cartilage outline was visible.

**Figure 2:**
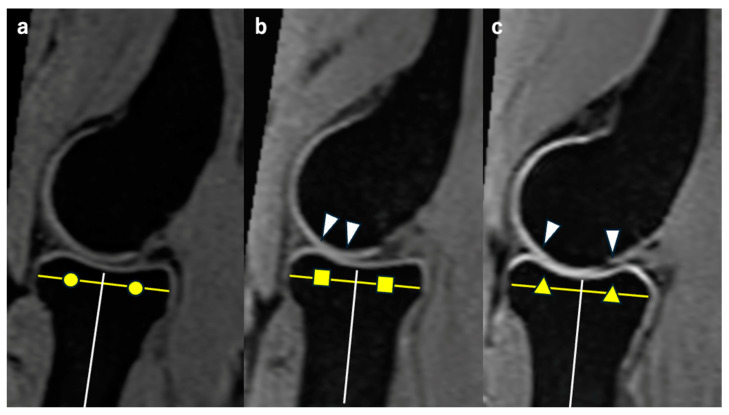
Representative MR sagittal images without traction from each age group. Each image passes through the midpoint of the radial head on the coronal image. We measured the JW on a vertical line extending proximally from the white line, which is the bisector of the radial head. The yellow lines represent the diameter of the radial head. Circles, squares and triangles in each figure represent that each white line divides the yellow line equally, making the white lines bisectors. (**a**) Representative image from the 20s. The HACV is classified as complete. (**b**) Representative image from the 30s. The HACV is classified as intermediate. The cartilage outline is not visible in the area between white arrowheads. (**c**) Representative image from the 40s. The HACV is classified as poor. The cartilage outline is not visible in the area between white arrowheads.

We evaluated the differences in HACV values with and without traction among different age groups (20s, 30s, and 40s) and assessed the effects of traction within each age group. Representative MR images of the RCJ without axial traction in each age group are shown in Figure 2.

### 2.7. The Pain and Discomfort Assessments

We also assessed pain and discomfort during MRI with and without traction. Each volunteer completed a visual analog scale questionnaire immediately after each MRI session to indicate whether axial traction had been applied. Volunteers rated their pain and discomfort on a scale of 0 to 10. The results of the questionnaire were then compiled.

We evaluated the differences in the values with and without traction across different age groups: 20s, 30s, and 40s. Additionally, we assessed changes in the values within each age group.

### 2.8. Statistical Analysis

Statistical analyses were performed using SPSS Statistics, version 29 (IBM Corp., Armonk, NY, USA). The intraclass correlation coefficient was used to calculate the intra- and interobserver correlations of the JSW assessments. We categorized the intraclass correlation coefficient values as follows: >0.8 indicated excellent correlation, 0.6–0.8 indicated good correlation, 0.4–0.6 indicated moderate correlation, 0.2–0.4 indicated fair correlation, and <0.2 indicated poor correlation [15]. Additionally, we used Cohen’s kappa (κ) statistic to evaluate the intra- and inter-observer reliabilities of the HACV [16]. The kappa values were graded as follows: >0.90 signified almost perfect agreement, 0.80–0.90 indicated strong agreement, 0.60–0.79 indicated moderate agreement, 0.40–0.59 indicated weak agreement, 0.21–0.39 indicated minimal agreement, and 0–0.20 indicated no agreement [17].

We assessed the normality of the data using the Shapiro–Wilk test, which revealed that none of the data followed a normal distribution. Consequently, we employed the Wilcoxon signed-rank test to determine the statistical significance of the differences in all assessments.

## 3. Results

In this study, we enrolled five males and five females in each age group: 20s, 30s, and 40s, among a total of 30 volunteers. The mean age was 34.6 ± 1.6 years (range 22–49). None of the volunteers had a history of an elbow injury. We present the demographic data of each volunteer in Table 1.

For the JW measurements, examiner 1’s intraobserver correlation coefficients were 0.99 for the RC, 0.95 for the LUH, and 0.93 for the MUH. Examiner 2 had intraobserver correlation coefficients of 0.98 for the RC, 0.85 for the LUH, and 0.86 for the MUH. The interobserver correlation coefficients for the JW measurements were 0.99 for the RC, 0.98 for LUH, and 0.97 for MUH. Overall, both examiners demonstrated excellent correlations for all measurements.

For HACV measurements, examiner 1’s intraobserver correlation coefficients were 0.92 for the RC, 0.94 for the LUH, and 0.95 for the MUH. Examiner 2 had intraobserver correlation coefficients of 0.91 for the RC, 0.86 for LUH, and 1.0 for MUH. The interobserver correlation coefficients for the HACV measurements were 0.97 for the RC, 0.88 for the LUH, and 0.83 for the MUH. Both examiners showed excellent agreement for all measurements.

These findings demonstrate the strong reproducibility of the measurement criteria. Consequently, we use examiner 1’s first measurement as representative data in the following section.

### 3.1. The JW in the RC

**Figure 3:**
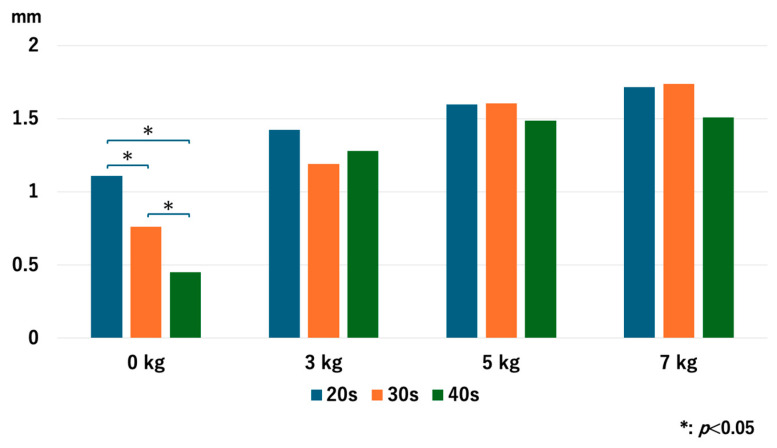
Comparison of the joint space width at the RC among different age groups. Older age groups had significantly narrower joint space width compared to the younger age groups. RC; radiocapitellar joint.

Without traction, the JW of the older age groups was significantly narrower than that of the younger age groups: *p* = 0.048 for 20s vs. 30s, *p* = 0.008 for 20s vs. 40s, and *p* = 0.008 for 30s vs. 40s. However, no significant differences were observed in the JW values after traction with each traction weight. With 3 kg of axial traction, *p* = 0.47 for 20s vs. 30s, *p* = 0.76 for 20s vs. 40s, and *p* = 0.77 for 30s vs. 40s. With 5 kg of axial traction, *p* = 0.98 for 20s vs. 30s, *p* = 0.81 for 20s vs. 40s, and *p* = 0.73 for 30s vs. 40s. With 7 kg of axial traction, *p* = 1.0 for 20s vs. 30s, *p* = 0.77 for 20s vs. 40s, and *p* = 0.56 for 30s vs. 40s (Figure 3).

**Figure 4:**
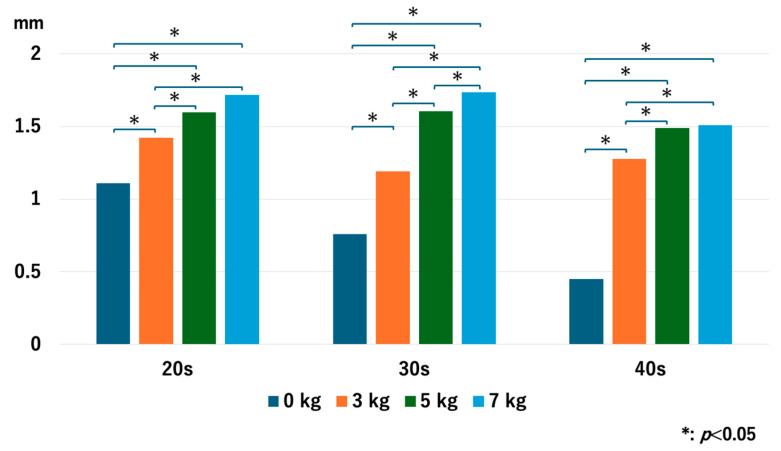
The joint space width of each age group at the RC. The joint space width significantly increased as we used heavier traction weight, except for 5 kg vs. 7 kg in the 20s and the 40s. RC; radiocapitellar joint.

For the 20s age group, the JW increased as heavier traction weights were used, but there was no significant difference between 5 kg and 7 kg: *p* = 0.013 for 0 kg vs. 3 kg, *p* = 0.005 for 0 kg vs. 5 kg, *p* = 0.005 for 0 kg vs. 7 kg, *p* = 0.028 for 3 kg vs. 5 kg, *p* = 0.009 for 3 kg vs. 7 kg, and *p* = 0.169 for 5 kg vs. 7 kg. For the 30s age group, JW increased with heavier traction weights: *p* = 0.008 for 0 kg vs. 3 kg, *p* = 0.005 for 0 kg vs. 5 kg, *p* = 0.005 for 0 kg vs. 7 kg, *p* = 0.005 for 3 kg vs. 5 kg, *p* = 0.005 for 3 kg vs. 7 kg, and *p* = 0.036 for 5 kg vs. 7 kg. For the 40s age group, JW increased with heavier traction weights, but there was no significant difference between 5 kg and 7 kg: *p* = 0.005 for 0 kg vs. 3 kg, *p* = 0.005 for 0 kg vs. 5 kg, *p* = 0.008 for 0 kg vs. 7 kg, *p* = 0.009 for 3 kg vs. 5 kg, *p* = 0.022 for 3 kg vs. 7 kg, and *p* = 0.721 for 5 kg vs. 7 kg (Figure 4). Interestingly, the older age groups had a narrower JW without traction, but the value of JW widening with 3 kg of traction increased as the age increased.

### 3.2. The HACV in the RC

**Figure 5:**
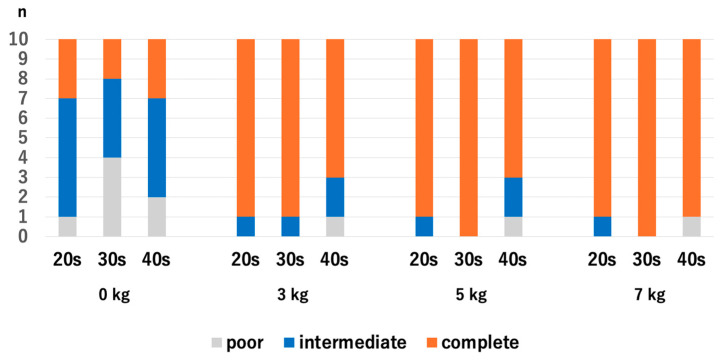
Comparison of the articular cartilage visibilities at the RC among different age groups. There were no significant differences. RC; radiocapitellar joint.

There were no significant differences in HACV among the age groups with and without traction. Without traction, the *p*-values were 0.218 for the 20s vs. 30s, 0.768 for 20s vs. 30s, and 0.372 for 30s vs. 40s. With a traction weight of 3 kg, the *p*-values were 1.0 for 20s vs. 30s, 0.255 for 20s vs. 40s, and 0.255 for 30s vs. 40s. With a traction weight of 5 kg, the *p*-values were 0.317 for the 20s vs. 30s, 0.255 for 20s vs. 30s, and 0.068 for 30s vs. 40s. With a traction weight of 7 kg, the *p*-values were 0.317 for 20s vs. 30s, 0.942 for 20s vs. 40s, and 0.317 for 30s vs. 40s (Figure 5).

**Figure 6:**
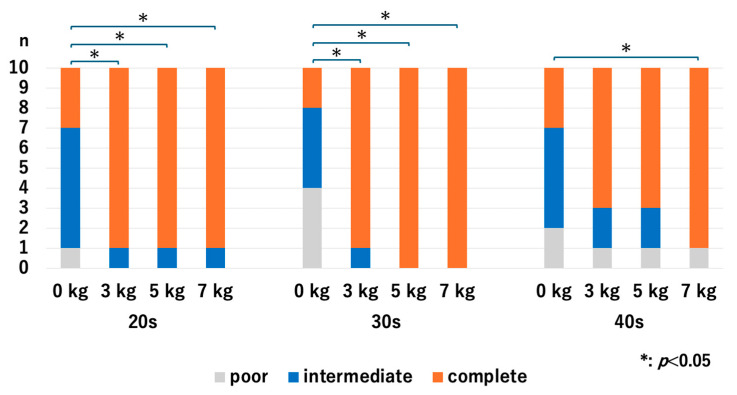
The articular cartilage visibilities of each age group at the RC. For the 40s, the only significant difference was between 0 kg and 7 kg. RC; radiocapitellar joint.

For the 20s age group, the HACV significantly improved with axial traction, but there were no significant differences among the traction weights we used: *p* = 0.007 for 0 kg vs. 3 kg, *p* = 0.007 for 0 kg vs. 5 kg, *p* = 0.007 for 0 kg vs. 7 kg, *p* = 1.0 for 3 kg vs. 5 kg, *p* = 1.0 for 3 kg vs. 7 kg, and *p* = 1.0 for 5 kg vs. 7 kg. For the 30s age group, the HACV significantly improved with axial traction, but there were no significant differences among the traction weights we used: *p* = 0.009 for 0 kg vs. 3 kg, *p* = 0.01 for 0 kg vs. 5 kg, *p* = 0.01 for 0 kg vs. 7 kg, *p* = 0.941 for 3 kg vs. 5 kg, *p* = 0.941 for 3 kg vs. 7 kg, and *p* = 1.0 for 5 kg vs. 7 kg. For the 40s age group, HACV significantly increased with the use of a 7 kg traction weight: *p* = 0.107 for 0 kg vs. 3 kg, *p* = 0.107 for 0 kg vs. 5 kg, *p* = 0.013 for 0 kg vs. 7 kg, *p* = 1.0 for 3 kg vs. 5 kg, *p* = 0.343 for 3 kg vs. 7 kg, and *p* = 0.343 for 5 kg vs. 7 kg (Figure 6). Even though the value of JW widening was the highest in the 40s with the application of 3 kg traction, significant improvement of the HACOV in the 40s was only seen with traction of 7 kg.

### 3.3. The JW in the LUH

**Figure 7:**
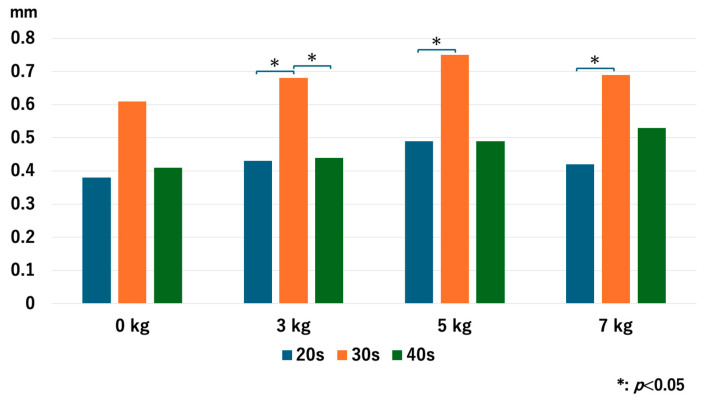
Comparison of the joint space width at the LUH among different age groups. With traction, the 30s group had significantly wider joint space width compared to the 20s group. LUH: lateral third of the ulnohumral joint.

Without traction, there were no significant differences among the age groups (*p* = 0.08 for 20s vs. 30s, *p* = 0.85 for 20s vs. 40s, and *p* = 0.10 for 30s vs. 40s). With axial traction, the JW for the 30s age group significantly increased compared to the 20s age group across all traction weights. However, when comparing the 30s and 40s age groups, a significant difference was only observed with 3 kg of axial traction. Specifically, the *p*-values for different traction weights are *p* = 0.03 for the 20s vs. 30s with 3 kg of traction, *p* = 0.94 for 20s vs. 40s, and *p* = 0.046 for 30s vs. 40s. With 5 kg of traction, the *p*-values were *p* = 0.049 for 20s vs. 30s, *p* = 0.62 for 20s vs. 40s, and *p* = 0.054 for 30s vs. 40s. For 7 kg of traction, the *p*-values were *p* = 0.417 for 20s vs. 30s, *p* = 1.0 for 20s vs. 40s, and *p* = 0.257 for 30s vs. 40s. (Figure 7).

**Figure 8:**
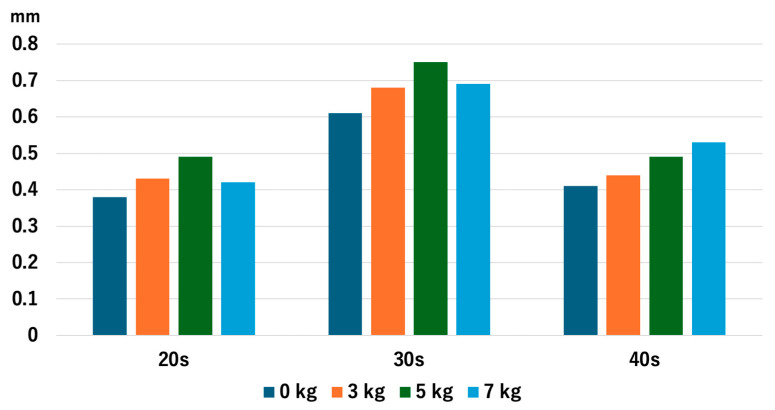
The joint space width of each age group at the LUH. The joint space width did not show significant change with and without traction. LUH: lateral third of the ulnohumral joint.

For all age groups, there were no significant differences in JW values among the traction weights used. For the 20s age group, the *p* values were 0.472 for 0 kg vs. 3 kg, 0.151 for 0 kg vs. 5 kg, 0.791 for 0 kg vs. 7 kg, 0.241 for 3 kg vs. 5 kg, 0.910 for 3 kg vs. 7 kg, and 0.307 for 5 kg vs. 7 kg. For the 30s age group, the *p*-values were 0.762 for 0 kg vs. 3 kg, 0.325 for 0 kg vs. 5 kg, 0.545 for 0 kg vs. 7 kg, 0.650 for 3 kg vs. 5 kg, 0.910 for 3 kg vs. 7 kg, and 0.571 for 5 kg vs. 7 kg. Lastly, for those in their 40s, the *p*-values were 0.880 for 0 kg vs. 3 kg, 0.495 for 0 kg vs. 5 kg, 0.256 for 0 kg vs. 7 kg, 0.545 for 3 kg vs. 5 kg, 0.384 for 3 kg vs. 7 kg, and 0.791 for 5 kg vs. 7 kg (Figure 8). The value of the JW in the 20s and 30s age groups decreased with traction of 7 kg, which was consistent with our previous study [14], while the value continuously increased in the 40s age group, although we observed no significant difference.

### 3.4. The HACV in the LUH

**Figure 9:**
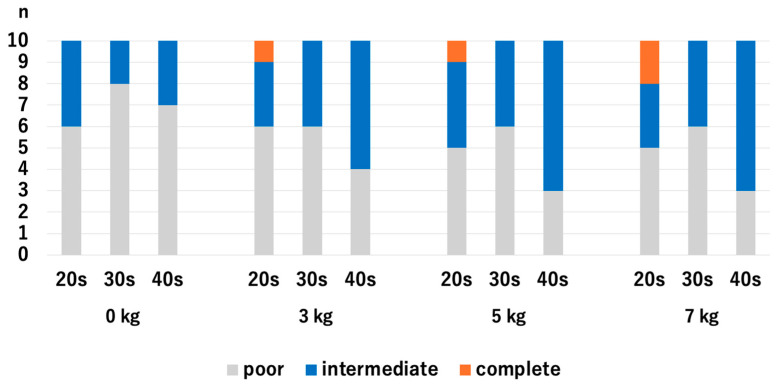
Comparison of the articular cartilage visibilities at the LUH among different age groups. There were no significant differences. LUH: lateral third of the ulnohumral joint.

There were no significant differences in HACV among the age groups with and without traction. Without traction, the *p*-values were 0.414 for the 20s vs. 30s, 0.655 for 20s vs. 40s, and 0.655 for 30s vs. 40s. With a traction weight of 3 kg, the *p*-values were 0.705 for 20s vs. 30s, 0.739 for 20s vs. 40s, and 0.414 for 30s vs. 40s. With a traction weight of 5 kg, the *p*-values were 0.564 for 20s vs. 30s, 0.655 for 20s vs. 40s, and 0.257 for 30s vs. 40s. With a traction weight of 7 kg, the *p*-values were 0.317 for the 20s vs. 30s, 0.942 for the 20s vs. 40s, and 0.317 for the 30s vs. 40s (Figure 9).

**Figure 10:**
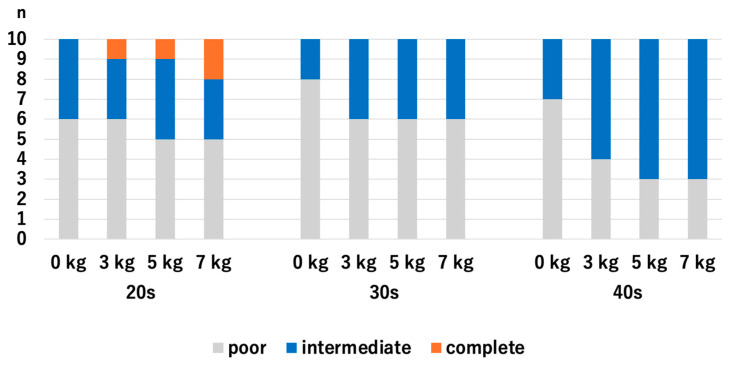
The articular cartilage visibilities of each age group at the LUH. There were no significant improvements in any of the age groups. LUH: lateral third of the ulnohumral joint.

For all age groups, there were no significant differences in HACV among the traction weights used. For the 20s age group, the *p*-values were 0.861 for 0 kg vs. 3 kg, 0.547 for 0 kg vs. 5 kg, 0.445 for 0 kg vs. 7 kg, 0.702 for 3 kg vs. 5 kg, 0.584 for 3 kg vs. 7 kg, and 0.836 for 5 kg vs. 7 kg. For the 30s age group, the *p*-values were 0.342 for 0 kg vs. 3 kg, 0.342 for 0 kg vs. 5 kg, 0.342 for 0 kg vs. 7 kg, 1.0 for 3 kg vs. 5 kg, 1.0 for 3 kg vs. 7 kg, and 1.0 for 5 kg vs. 7 kg. For the 40s age group, the *p*-values were 0.189 for 0 kg vs. 3 kg, 0.081 for 0 kg vs. 5 kg, 0.081 for 0 kg vs. 7 kg, 0.648 for 3 kg vs. 5 kg, 0.648 for 3 kg vs. 7 kg, and 1.000 for 5 kg vs. 7 kg (Figure 10). This trend was consistent with our previous study [14].

### 3.5. The JW in the MUH

**Figure 11:**
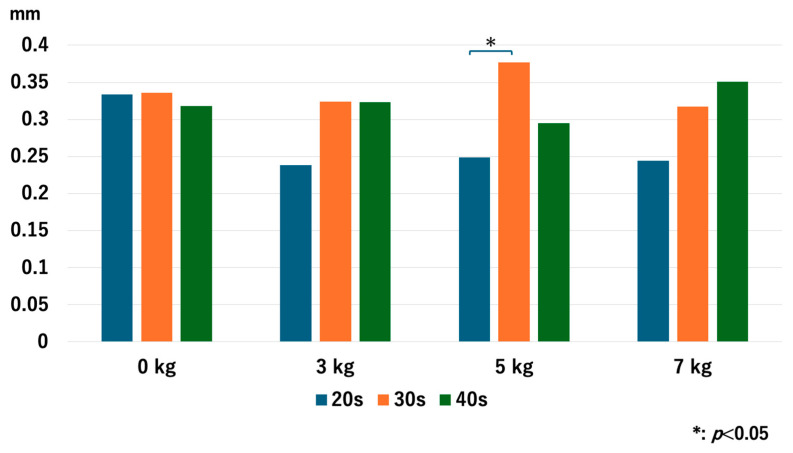
Comparison of the joint space width at the MUH among different age groups. With traction of 5 kg, the 30s had significantly wider joint space width compared to the 20s. MUH: medial third of the ulnohumral joint.

Without traction, there were no significant differences in JW among the age groups, with *p*-values of 0.72 for the comparison between the 20s and 30s age groups, 0.84 for the 20s vs. 40s, and 0.88 for the 30s vs. 40s. The only significant difference observed was observed between 20s and 30s age groups when 5 kg of axial traction were applied, with a *p*-value of 0.014. For 3 kg of axial traction, the *p*-values were 0.28 for the 20s vs. 30s, 0.36 for the 20s vs. 40s, and 0.96 for the 30s vs. 40s. With 5 kg of axial traction, the *p*-values were 0.014 for the 20s vs. 30s, 0.55 for the 20s vs. 40s, and 0.26 for the 30s vs. 40s. With 7 kg of axial traction, the *p*-values were 0.26 for the 20s vs. 30s, 0.31 for the 20s vs. 40s, and 0.80 for the 30s vs. 40s (Figure 11).

**Figure 12:**
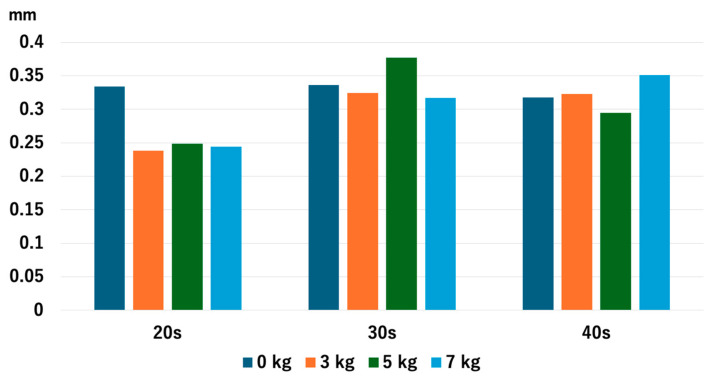
The joint space width of each age group at the MUH. There were no significant differences observed. MUH: medial third of the ulnohumral joint.

Across all age groups, there were no significant differences in JW values with the application of different traction weights. For individuals in their 20s, the *p*-values indicated no significant changes across comparisons between 0 kg and 3 kg, 5 kg, or 7 kg, as well as between the traction weights themselves. Similarly, participants in their 30s and 40s showed no significant differences in JW values regardless of the traction weight used, highlighting that varying traction weights did not significantly impact the JW values within any age group (Figure 12). The differences in the value of the JW in the MUH were minimal throughout all the age groups and the application of traction, which was consistent with our previous study [14].

### 3.6. The HACV in the MUH

**Figure 13:**
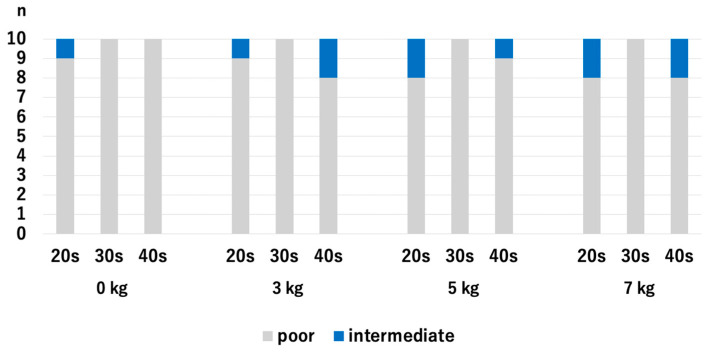
Comparison of the articular cartilage visibilities at the MUH among different age groups. There were no significant differences. MUH: medial third of the ulnohumral joint.

There were no significant differences in HACV among the age groups with and without traction. Without traction, *p*-values were 0.317 for the 20s vs. 30s, 0.317 for 20s vs. 40s, and 1.0 for the 30s vs. 40s. With a traction weight of 3 kg, the *p*-values were 0.317 for 20s vs. 30s, 0.542 for 20s vs. 40s, and 0.146 for 30s vs. 40s. With a traction weight of 5 kg, the *p*-values were 0.146 for 20s vs. 30s, 0.146 for 20s vs. 40s, and 0.317 for 30s vs. 40s. With a traction weight of 7 kg, the *p*-values were 0.146 for the 20s vs. 40s, 1.0 for the 20s vs. 40s, and 0.146 for the 30s vs. 40s (Figure 13).

**Figure 14:**
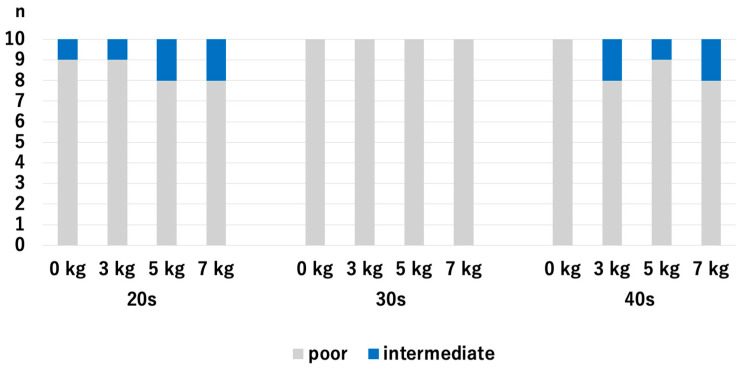
The articular cartilage visibilities of each age group at the MUH. There were no significant improvements in any of the age groups. MUH: medial third of the ulnohumral joint.

Across all age groups, no significant differences were found in JW values among the traction weights used. In the 20s group, *p*-values were 1.0 for 0 kg vs. 3 kg, 0.542 for 0 kg vs. 5 kg, 0.542 for 0 kg vs. 7 kg, 0.542 for 3 kg vs. 5 kg, 0.542 for 3 kg vs. 7 kg, and 1.0 for 5 kg vs. 7 kg. For the 30s group, the *p*-values were 0.146 for 0 kg vs. 3 kg, 0.317 for 0 kg vs. 5 kg, 0.146 for 0 kg vs. 7 kg, 0.542 for 3 kg vs. 5 kg, 1.000 for 3 kg vs. 7 kg, and 0.542 for 5 kg vs. 7 kg. For the 40s group, the *p*-values were 0.146 for 0 kg vs. 3 kg, 0.317 for 0 kg vs. 5 kg, 0.146 for 0 kg vs. 7 kg, 0.542 for 3 kg vs. 5 kg, 1.0 for 3 kg vs. 7 kg, and 0.542 for 5 kg vs. 7 kg (Figure 14). This trend was consistent with our previous study [14].

### 3.7. The Pain

**Figure 15:**
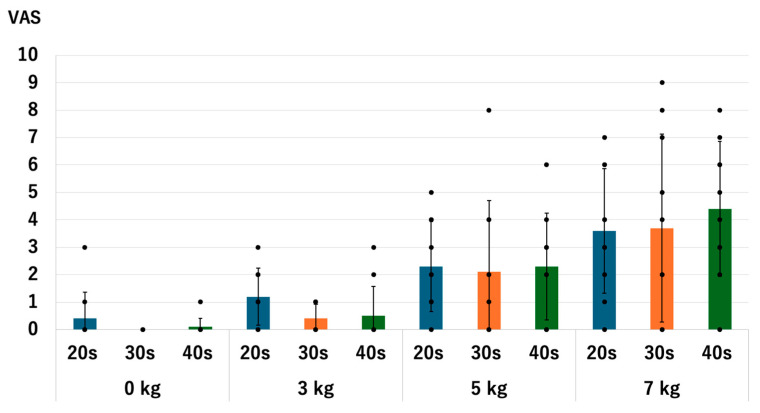
Comparison of the pain scores among different age groups. Each dot represents the actual data. There were no significant differences observed. VAS: visual analogue scale.

There were no significant differences in the level of pain between age groups with and without traction. Without traction, the *p*-values were 0.679 for 20s vs. 30s, 0.414 for 20s vs. 40s, and 0.317 for the 30s vs. 40s. With a traction weight of 3 kg; the *p*-values were 0.052 for 20s vs. 30s, 0.307 for 20s vs. 40s, and 0.679 for 30s vs. 40s. With a traction weight of 5 kg, *p*-values were 0.763 for 20s vs. 30s, 1.0 for 20s vs. 40s, and 1.0 for 30s vs. 40s. With a traction weight of 7 kg, the *p*-values were 0.953 for the 20s vs. 40s, 0.413 for the 20s vs. 40s, and 0.526 for the 30s vs. 40s (Figure 15).

**Figure 16:**
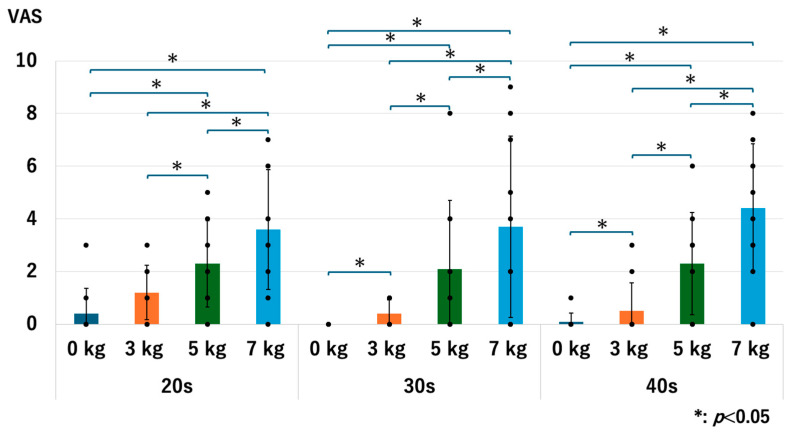
The pain scores of each age group. Each dot represents the actual data. The pain increased as we used the heavier traction weights, except for 0 kg vs. 3 kg in the 20s and 40s groups. VAS: visual analogue scale.

For all age groups, the pain value significantly increased when a heavier traction weight was used, except for the comparison between 0 kg vs. 3 kg in the 20s and 40s groups. For the 20s group, the *p*-values were 0.071 for 0 kg vs. 3 kg, 0.017 for 0 kg vs. 5 kg, 0.013 for 0 kg vs. 7 kg, 0.015 for 3 kg vs. 5 kg, 0.016 for 3 kg vs. 7 kg, and 0.020 for 5 kg vs. 7 kg. For the 30s group, the *p*-values were 0.046 for 0 kg vs. 3 kg, 0.027 for 0 kg vs. 5 kg, 0.018 for 0 kg vs. 7 kg, 0.027 for 3 kg vs. 5 kg, 0.018 for 3 kg vs. 7 kg, and 0.027 for 5 kg vs. 7 kg. For the 40s group, the *p*-values were 0.071 for 0 kg vs. 3 kg, 0.017 for 0 kg vs. 5 kg, 0.007 for 0 kg vs. 7 kg, 0.017 for 3 kg vs. 5 kg, 0.007 for 3 kg vs. 7 kg, and 0.007 for 5 kg vs. 7 kg (Figure 16). We observed the same trend in our previous studies [13,14].

### 3.8. The Discomfort

**Figure 17:**
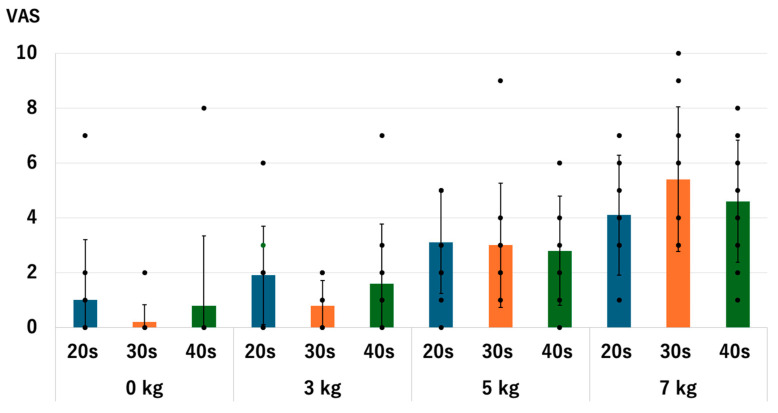
Comparison of the discomfort scores among different age groups. Each dot represents the actual data. There were no significant differences observed. VAS: visual analogue scale.

There were no significant differences in discomfort between age groups with and without traction. Without traction, the *p*-values were 0.357 for the 20s vs. 30s, 0.715 for 20s vs. 40s, and 0.314 for the 30s vs. 40s. With a traction weight of 3 kg, the *p*-values were 0.066 for 20s vs. 30s, 0.478 for 20s vs. 40s, and 0.395 for 30s vs. 40s. With a traction weight of 5 kg, the *p*-values were 0.832 for 20s vs. 30s, 0.673 for 20s vs. 40s, and 0.833 for 30s vs. 40s. With a traction weight of 7 kg, the *p*-values were 0.217 for the 20s vs. 40s, 0.553 for the 20s vs. 40s, and 0.289 for the 30s vs. 40s (Figure 17).

**Figure 18:**
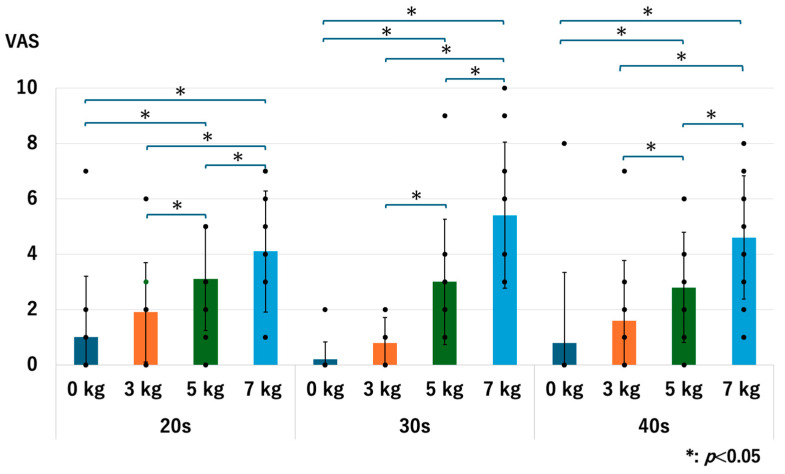
The discomfort scores of each age group. Each dot represents the actual data. The pain increased as we used the heavier traction weights, except for 0 kg vs. 3 kg in all age groups. VAS: visual analogue scale.

Across all age groups, discomfort levels significantly increased with heavier traction weights, although no significant differences were observed between 0 kg and 3 kg in any age group. In the 20s age group, discomfort significantly rose with the use of 5 kg and 7 kg of traction compared to 0 kg, and differences between 3 kg and 5 kg, as well as 3 kg and 7 kg, were also significant. For the 30s age group, discomfort levels significantly increased with 5 kg and 7 kg of traction compared to 0 kg, with notable increases also observed between 3 kg and 5 kg and 3 kg and 7 kg. In the 40s age group, the discomfort significantly increased with 5 kg and 7 kg of traction compared to 0 kg, and differences between 3 kg and 5 kg, as well as 3 kg and 7 kg, were also significant (Figure 18). We observed the same trend in our previous studies [13,14].

## 4. Discussion

We evaluated the effect of axial traction on elbow MRI scans in three different age groups. Our results indicated that without traction, the older age groups exhibited significantly narrower JW at the RC, whereas no significant differences were observed at the LUH and the MUH. Aging is a well-known risk factor for OA [18,19], which is characterized by a progressive narrowing of the joint space [20]. This narrowing is attributed to mechanical loads on a joint that exceed its repair capacity, leading to degeneration of articular cartilage, subchondral bone, and supporting tissues such as capsules, ligaments, and tendons [20]. Specifically, at the elbow, OA is thought to initially affect the RC before impacting the LUH [21]. These findings align with our results, which show that older age groups have a narrower JW at the RC, consistent with the degenerative changes associated with OA. At the RC, JW increased with heavier traction weights, except for 5 kg and 7 kg traction in the 20s and 40s age groups, respectively. These findings are consistent with that of our previous study [14]. Additionally, JW after traction did not differ significantly among different age groups, regardless of the traction weights used. Regarding the HACV, the 20s and 30s age groups showed significant improvements with all traction weights. In the 40s, HACOV also improved with traction, with the proportion of complete visibility being 30% without traction, 70% with 3 kg and 5 kg of axial traction, and 90% with 7 kg of axial traction. However, a significant difference in HACV in the 40s group was observed between 0 kg and 7 kg. In other words, while axial traction is effective for joint space widening, a heavier traction weight is necessary for improving HACV in the 40s age group.

It is important to note that we measured JW at the center of the RC; thus, joint space widening may have been insufficient at other points of the RC in the 40s age group. Joint stiffness, which increases as joint degeneration progresses [20], may explain this result. Considering these findings, for the 20s and 30s, axial traction of 3 kg was effective in improving articular cartilage visibility, as noted in our previous study [14]. For patients in their 40s, we recommend using a traction weight of 7 kg to enhance articular cartilage visibility, as 3 kg of traction may not provide sufficient improvement in HACV for this age group.

At LUH and MUH, axial traction did not increase JW or improve HACV. These results align with our previous studies [13,14]. At the LUH, the JW in the 20s and 30s age groups initially widened with heavier traction weights up to 5 kg but then narrowed with 7 kg of traction. In contrast, for the 40s age group, the JW of the LUH slightly increased with heavier traction weights.

In a previous study [14], we hypothesized that the strong medial collateral ligament of the elbow [22,23] during axial traction causes the elbow to function as a loose hinge joint with screw displacement movement, pivoting around the medial epicondyle [24]. For individuals in their 20s and 30s, applying 7 kg of axial traction might have overextended the RC, realigning the elbow from valgus to varus, which could explain the narrowing of the JW at the LHU [14]. For the 40s age group, due to tissue degeneration, the 7 kg traction might have been insufficient to overextend the RC, resulting in the observed minimal changes.

The causes of elbow pain vary depending on the patient’s age. Trauma and sports-related injuries such as osteochondritis dissecans are more common in younger age groups. Contrastingly, the proportion of people who present with chronic elbow pain, including osteoarthritis of the elbow, increases in older age groups. Therefore, even if the MR imaging aims to evaluate the articular cartilage, the target disease will likely differ depending on each patient’s age. If an image evaluation of the articular cartilage of the elbow joint is required in patients over 40 who have no traumatic episodes, the possibility of osteoarthritis of the elbow cannot be excluded. To perform a sufficient evaluation, performing an MRI with 7 kg of traction may be useful, considering the results of this study. The participants in this study were healthy individuals, and it was confirmed that there were no symptoms such as limited elbow joint range of motion or pain prior to the imaging. However, considering that the JW without traction was narrower with age, it is possible that participants with non-symptomatic osteoarthritis were included. The usefulness of 7 kg of traction for patients with symptomatic osteoarthritis of the elbow has not yet been evaluated, and further investigation is required.

The mean values of pain and discomfort increased with heavier traction weights, but there were no significant differences among the different age groups. No statistical differences were observed between 0 kg and 3 kg of traction in any age group, except for pain in the 30s age group. For this group, the mean pain value with 3 kg of traction was 0.4, which supports our previous finding that 3 kg of axial traction is relatively safe. For individuals in their 40s, the mean values for pain and discomfort with a traction weight of 7 kg were 4.4 and 4.6, respectively. This indicates that those in their 40s need to manage increased pain and discomfort to achieve better visibility of articular cartilage, highlighting a challenge that must be addressed. In clinical settings, when traction MRI of the elbow was indicated in patients over 40, an effective approach to decrease pain and discomfort in patients can be the administration of painkillers such as non-steroid anti-inflammatory drugs several hours prior to the imaging. However, further investigation is necessary to determine the optimal approach.

This study has several limitations. First, the sample size was small, which limited the study’s statistical power. The narrow age range (20s, 30s, and 40s) also restricted the generalizability of the findings. Volunteers were recruited from our hospital’s working staff, making it challenging to include a broader population, particularly older adults over 50. In the future, we will investigate and clarify the effect of axial traction on MRI of the elbow with participants over 50 years of age. We noted a slightly wider JW at the LUH in the 30s group compared to the other age groups, with and without traction. However, this difference was minimal (less than 0.5 mm) and did not impact the HACV, likely due to the characteristics of the volunteers and the small sample size. Additionally, as a single-center study involving volunteers, our findings may not be broadly applicable. Future research should consider a multicenter approach with a more diverse population. In our previous study, we explored criteria for traction MRI of the elbow based on body size, age, and sex and gathered some evidence regarding age effects. However, further research is needed to assess the impact of axial traction on patients with varied characteristics across a wider age range.

## 5. Conclusions

As hypothesized, the effect of axial traction at the RC in elbow MRI decreased in patients in their 40s. For patients in their 20s and 30s, a traction weight of 3 kg is generally sufficient to improve articular cartilage visibility of the RC. However, for individuals in their 40s who do not achieve adequate articular cartilage visibility with 3 kg of axial traction, we recommend applying a traction weight of 7 kg. We propose that the results obtained in this study can be used as standard reference values for healthy individuals. We will investigate a wider range of age groups, including participants in their 50s and older, to further clarify the effectiveness of traction MRI of the elbow. Additionally, we plan to conduct studies involving actual patients to clarify the clinical implications of the procedure.

## Figures and Tables

**Table 1:** Demographic data of the volunteers.

20s		30s		40s	
Sex	Age	Sex	Age	Sex	Age
F	22	F	30	M	41
F	23	F	32	F	43
M	23	M	32	F	43
M	23	F	33	M	43
F	25	F	33	F	45
M	26	M	33	M	45
M	26	F	34	F	46
F	28	M	34	M	46
M	28	M	38	F	48
F	29	M	38	M	49

## Data Availability

Data are contained within the article.
